# Supplementary material for: Application of Oxidative Stress to a Tissue-Engineered Vascular Aging Model Induces Endothelial Cell Senescence and Activation
Source: Cells. 2020 May 22;9(5):1292. doi: 10.3390/cells9051292 (PMC7290800; doi:10.3390/cells9051292)
Supplement: Supplementary file 1 [file cells-09-01292-s001.pdf]

cells

MDPI

Supplementary Materials

Application of Oxidative Stress to a Tissue-Engineered Vascular Aging Model Induces Endothelial Cell Senescence and Activation

Ellen E. Salmon [1], Jason J. Breithaupt [2] and George A. Truskey [1,*]

[1] Department of Biomedical Engineering, Duke University, Durham, NC 27708; ellen.salmon@duke.edu
[2] Miller School of Medicine, University of Miami, Miami, FL 33136; jjb239@miami.edu
* Correspondence: gtruskey@duke.edu; Tel.: +01-919-660-5147

Received: 2 May 2020; Accepted: 18 May 2020; Published: date

Table 1. Cord Blood ECFC Characterization Antibodies.

Blood ECFC Characterizatio	Number 1 Antibodies	Marker Result Antibody
CD 31	303,103	Positive
CD 34	343,603	Positive
CD 144	348,505	Positive
CD 105	323,206	Positive
CD 14	325,603	Negative
CD 45	304,005	Negative
CD 115	347,303	Negative
Mouse IgG1	400,108	Control
Mouse IgG1	400,112	Control

1 All antibodies were purchased from BioLegend.

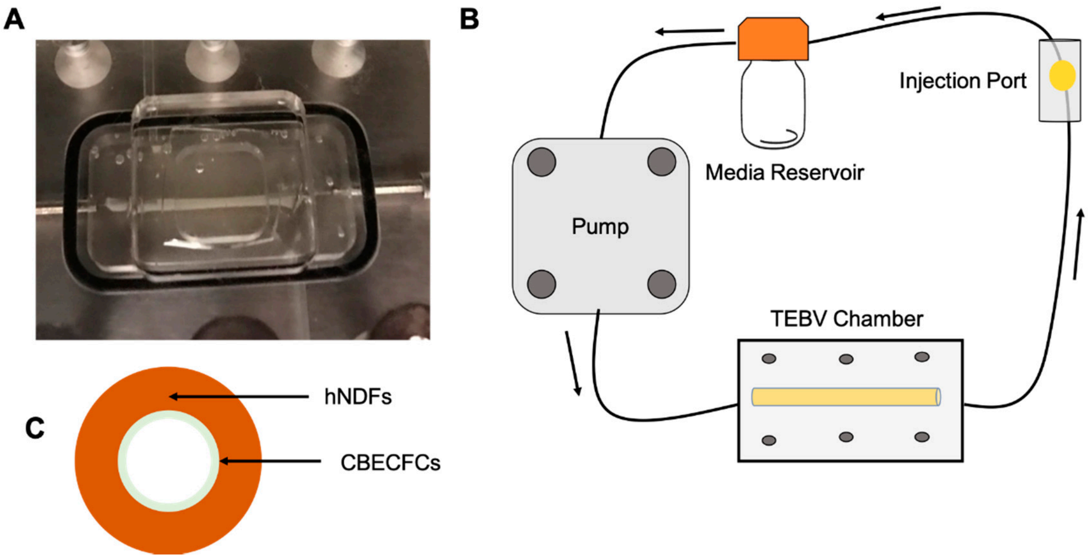

Figure S1. TEBV Images and Flow Circuit Schematic. (**A**) An image of a complete TEBV mounted in the perfusion chamber. The TEBV displayed was imaged in phenol-free LG DMEM for clearer visualization. The microscope cover slide embedded in the center of the acrylic chamber allows for higher quality imaging of the sample without compromising sterility or the integrity of the chamber at the required pressures. (**B**) A diagram of the TEBV flow circuit. Media flows from the reservoir through the pump, then into the TEBV chamber. The injection port used for drug studies is in between

the chamber and the reservoir so that bolus injections may mix with the bulk of the media before reaching the TEBV. (**C**) A cross-sectional representation of the TEBV. The hNDF-embedded gel comprises the bulk of the volume of the TEBV. The endothelium is present in a monolayer on the lumen of the vessel.

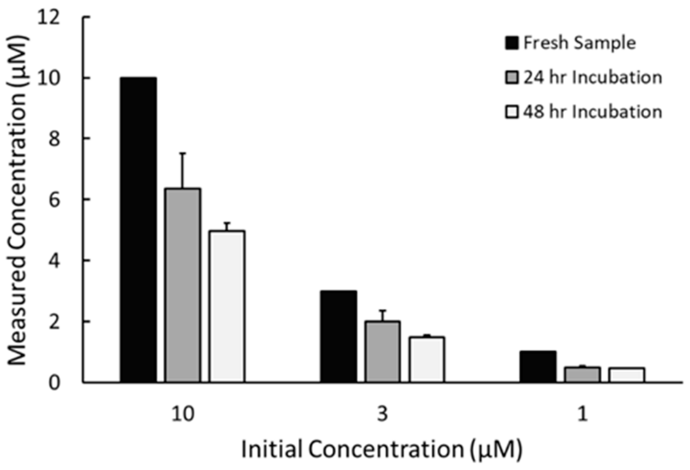

Figure S2. Degradation of H_2O_2in solution over time. 10, 3, and 1 µM solutions of H_2O_2were prepared in assay buffer and left at 37 °C for up to 48 h. The samples were further diluted in assay buffer at the time of analysis to samples within the detection limit of the assay and the analyzed at 3 points along the standard curve. These were compared to freshly prepared samples on the standard curve. Higher concentrations of H_2O_2 could not be used due to the low maximum threshold of the assay. The cell culture media used for experiments could not be used, but the degradation of H_2O_2 in the two solutions is expected to be comparable.

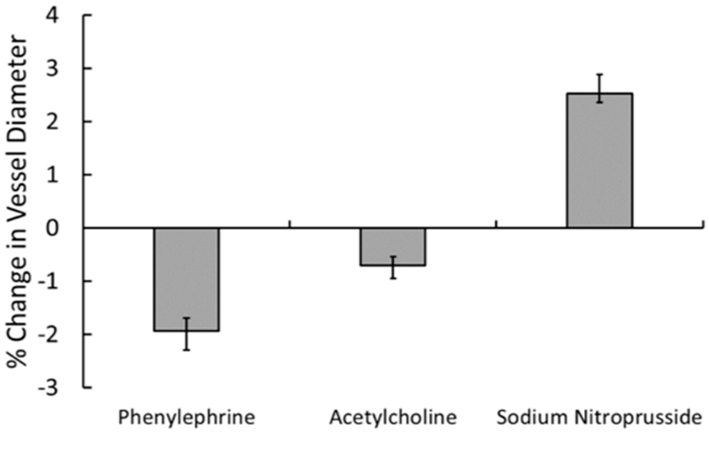

Figure S3. Vasoreactivity of hNDF-only TEBVs. TEBVs fabricated without an endothelium and matured for 7 days under standard perfusion conditions exhibit the same vasoconstriction as endothelialized vessels on Day 7. Like vessels treated with 100 µM H_2O_2, there is slight constriction in response to acetylcholine in the absence of a functional endothelium. However, vasodilation in response to sodium nitroprusside still occurs. Data represent mean ± SEM. N = 4.

© 2020 by the authors. Submitted for possible open access publication under the terms and conditions of the Creative Commons Attribution (CC BY) license (http://creativecommons.org/licenses/by/4.0/).
